# Retinal pigment epithelial cell expression of active Rap 1a by scAAV2 inhibits choroidal neovascularization

**DOI:** 10.1038/mtm.2016.56

**Published:** 2016-08-24

**Authors:** Haibo Wang, Xiaokun Han, Colin A Bretz, Silke Becker, Deeksha Gambhir, George W Smith, R Jude Samulski, Erika S Wittchen, Lawrence A Quilliam, Magdalena Chrzanowska-Wodnicka, M Elizabeth Hartnett

**Affiliations:** 1 The John Moran Eye Center, University of Utah, Salt Lake City, Utah, USA; 2 Department of Ophthalmology, The Fourth Affiliated Hospital of China Medical University, Eye Hospital of China Medical University, Key Lens Research Laboratory of Liaoning Province, Shenyang, P.R. China; 3 UNC Vector Core, University of North Carolina at Chapel Hill, Chapel Hill, North Carolina, USA; 4 Department of Cell Biology and Physiology, University of North Carolina at Chapel Hill, Chapel Hill, North Carolina, USA; 5 Department of Biochemistry and Molecular Biology, Indiana University School of Medicine, Indianapolis, Indiana, USA; 6 Blood Research Institute, Blood Center of Wisconsin, Milwaukee, Wisconsin, USA

## Abstract

To test the hypothesis that increased Rap1a activity specifically in retinal pigment epithelial cells resists choroidal neovascularization (CNV), self-complementary adeno-associated virus 2 (scAAV2) with RPE65-promoter-driven GFP vectors were generated and introduced subretinally into *Rap1b*-deficient mice. Six-week-old mice that received subretinal control (scAAV2-Con) or constitutively active Rap1a (scAAV2-CARap1a) showed strong GFP at the 5 × 10^8^ viral particle/µl dose 5 weeks later without altering retinal morphology or function. Compared to scAAV2-Con- or phosphate-buffered saline (PBS)-injected, eyes injected with scAAV2-CARap1a had increased Rap1 in retinal pigment epithelial (RPE)/choroidal lysates and a significant reduction in CNV volume 7 days after laser, comparable to eyes that received intravitreal anti-VEGF versus IgG control. scAAV2-CARap1a-, but not anti-VEGF-, injected eyes had increased pan-cadherin in RPE/choroids. In cultured RPE cells, increased active Rap1a inhibited TNFα-induced disassociation of junctional pan-cadherin/β-catenin complexes, increased transepithelial electrical resistance through an interaction of β-catenin with phosphorylated scaffold protein, IQGAP1, and inhibited choroidal endothelial cell (CEC) transmigration of an RPE monolayer. This evidence shows that increased Rap1a activity specifically in RPE cells is sufficient to reduce CEC transmigration and CNV and involves IQGAP1-mediated protection of RPE junctional complexes.

## Introduction

Neovascular age-related macular degeneration (AMD) is a leading cause of legal blindness in the elderly.^1,2^ There are a number of reasons for vision loss, but the most common involve compromise of the retinal pigment epithelial (RPE) barrier integrity, activation of choroidal endothelial cells (CECs) to migrate across the RPE monolayer into the sensory retina, and the development of choroidal neovascularization (CNV). The RPE monolayer creates the “outer blood retinal barrier”, but with aging and other stresses, the barrier integrity of the RPE monolayer becomes compromised.^3^ RPE barrier integrity requires integration of junctional proteins that make up adherens and tight junctions.^4^ The junctional protein complex of the RPE barrier disassembles with physiologic stresses and can be restored.^5^ However, with repeated insults, crosstalk between signaling pathways, or overwhelming pathologic stresses, barrier compromise occurs.^6^ There is evidence that RPE barrier integrity is necessary to contain angiogenic growth factors and fluid from the choroidal vasculature beneath the basal aspect of the RPE monolayer^7^ and restrict CEC transmigration^8^ into the sensory retina, whereas reduced RPE barrier integrity is speculated to allow growth factor and fluid movement and facilitate CEC transmigration into the sensory retina.^9^ Clinical studies support the line of thinking that RPE barrier integrity reduces vision loss from neovascular AMD.^10^ Therefore, a means to maintain or restore RPE barrier integrity may prevent or reduce vision loss from neovascular AMD.

Rap1 is a GTPase of the Ras superfamily that is important in cell junction maintenance^11^ and motility.^12^ Rap1 has two isoforms, Rap1a and Rap1b, which are encoded on different chromosomes but have 95% homology.^13^ For either Rap1 isoform to cause a biologic event, the GTPase must be activated by a guanine nucleotide exchange factor (GEF), stimulating the binding of GTP and subsequent activation of downstream effector proteins. A GTPase-activating protein (GAP) then renders the GTPase inactive by aiding the intrinsic rate of hydrolysis of GTP back to GDP. Thus, Rap1 isoforms act as biologic switches, cycling between active and inactive forms. In cultured RPE cells, activation of each isoform increased RPE junctional integrity by different mechanisms. Rap1a restricted RPE junction disassembly, whereas Rap1b facilitated junctional reassembly.^9^ The mechanism of the effect of Rap1a involved interference with the assembly of NADPH oxidase subunits and with NADPH oxidase-generated reactive oxygen species (ROS). NADPH oxidase-generated ROS phosphorylate cadherins and disrupt adherens junctional complexes of the RPE monolayer.^14^ Rap1a interacted with NADPH oxidase subunit, p22phox, and prevented the interaction of p22phox with other subunits needed to activate NADPH oxidase.^14^ Active Rap1a also reduced CEC transmigration of an RPE monolayer.^14^ Therefore, activation of Rap1a in cultured RPE cells limited pathologic events that are important in the development of neovascular AMD. Since Rap1b is involved in angiogenesis,^15,16^ activation of both isoforms might stimulate angiogenesis through active Rap1b and be counterproductive in restricting CNV through active Rap1a, depending on the prevalence and activation of Rap1 isoforms.

In this study, we tested the hypothesis that active Rap1a introduced specifically into RPE cells would resist laser induced CNV in an *in vivo* model and probed the mechanisms involved in the activation of Rap1a on RPE barrier integrity.

## Results

### Generation of self-complementary adeno-associated virus 2 (scAAV2) driven by RPE65 promoter to deliver active Rap1a to RPE cells

**Figure 1:**
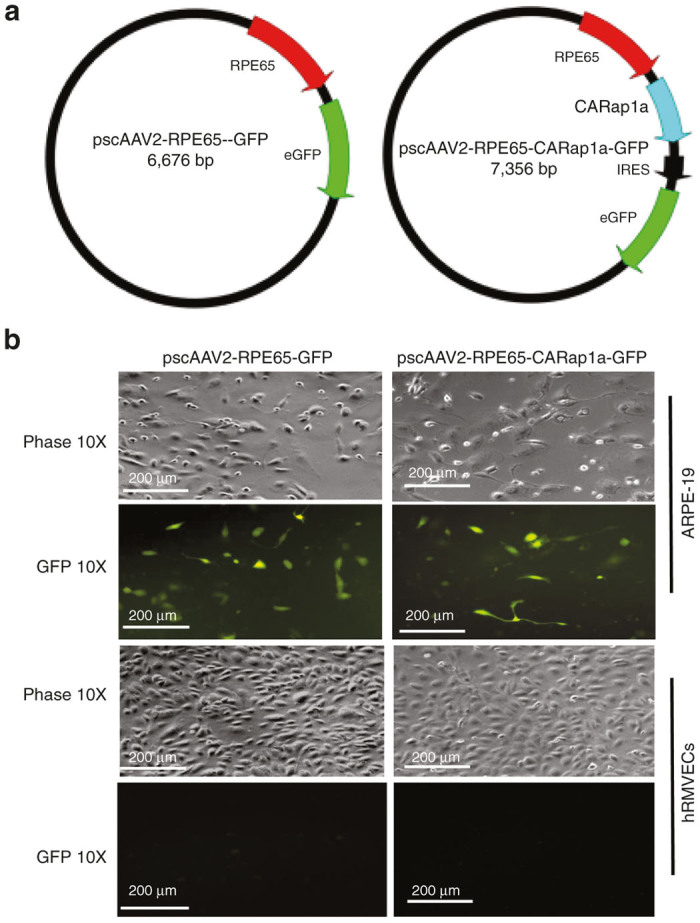
Generation of self-complementary adeno-associated virus 2 (scAAV2) to deliver constitutively active Rap1a (CARap1a) driven by an RPE65 promoter to retinal pigment epithelial (RPE) cells. (**a**) Diagrams of the scAAV2 containing the RPE cell-specific *RPE65* promoter driving GFP (control vector scAAV2-RPE65-GFP) or CARap1a (scAAV2-RPE65-CARap1a-GFP); (**b**) images taken under fluorescent microcopy showing GFP expression in a cell line of human RPE cells, ARPE-19, but not in human retinal microvascular endothelial cells (hRMVECs), each transfected with plasmid DNA of scAAV2-RPE65-GFP or scAAV2-RPE65-CARap1a-GFP.

To address the hypothesis that activation of Rap1a specifically in RPE cells would reduce the formation of CNV *in vivo*, we first generated self-complementary adeno-associated virus 2 (scAAV2) with a green fluorescent protein (GFP) tag, which contains a murine RPE65 promoter that specifically targets RPE cells (kindly provided by T. Michael Redmond, PhD). A synthetic coding sequence for the point mutation, constitutively active human Rap1a Q63E mutant (CARap1a)^17^ was later cloned into the scAAV2 as pscAAV2-RPE65-CARap1a-GFP, and the scAAV2 vector driving GFP only (pscAAV2-RPE65-GFP) was used as a control vector (Figure 1a). The active Rap1a amino acid sequences of murine and human are identical. To test RPE cell-specific targeting of scAAV2 vectors, scAAV2 plasmid DNAs expressing GFP or CARap1a were transfected into ARPE-19, a cell line of human RPE cells, or human retinal microvascular endothelial cells (hRMVECs), and expression of GFP was used to assess plasmid DNA expression. As shown in Figure 1b, GFP was visualized in ARPE-19 cells, but not hRMVECs, indicating that scAAV2 vectors driven by the RPE65 promoter specifically targeted RPE cells *in vitro*. Although the promoter was a murine RPE65, it can drive some GFP expression in human cells (T. Michael Redmond, personal communication, 2016).

### *In vivo* analysis of scAAV2 transduction and Rap1 expression in RPE cells and the effect of scAAV2 on retinal structure and visual function

**Figure 2:**
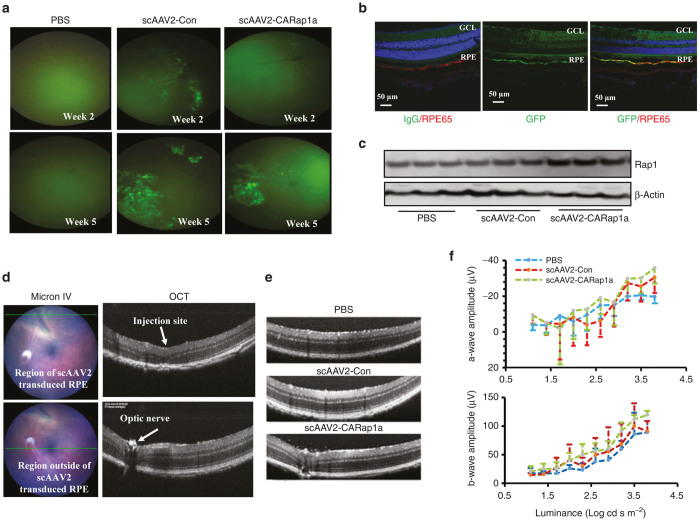
*In vivo* analysis of scAAV2 transduction in RPE cells of mice injected with scAAV2 vectors at dose of 5 × 10^8^ viral particle/µl. (**a**) Micron IV retinal imaging of GFP and (**b**) Immunostaining of GFP or isotype IgG and RPE65 in retinal/ retinal pigment epithelial (RPE)/choroidal cryosections in scAAV2 or PBS-injected *wild type* mice; (**c**) western blots of Rap1 protein in RPE/choroids from scAAV2 or PBS injected *Rap1b*^*+/-*^ mice; (**d**) Optical coherence tomography (OCT) of retinal structure and morphology directed by Micron IV retinal imaging; (**e**) OCTs through regions of previous subretinal injections and (**f**) Focal electroretinography (ERG) of scAAV2 or PBS-injected *Rap1b*^*+/-*^ mice prior to laser injury.

We next determined whether the scAAV2 viral vectors efficiently and specifically targeted RPE cells *in vivo*. Plasmid DNA scAAV2-RPE65-CARap1a-GFP or scAAV2-RPE65-GFP was used to produce experimental or control scAAV2 virus, named scAAV2-CARap1a or scAAV2-Con, respectively. Either scAAV2-CARap1a or scAAV2-Con at different doses was administered via subretinal injection into both eyes of 6-week-old wild-type C57Bl/6 mice (WT) mice. An injection caused a transient retinal detachment that affected about a third of the posterior eye and enabled transduction of RPE. An additional group of mice received bilateral subretinal injections of PBS as a control for the scAAV2 viral injections. Viral transduction was monitored by GFP expression using the Micron IV retinal imaging system weekly from week 2 to week 5. As shown in Figure 2, both scAAV2-CARap1a and scAAV2-Con at 5 × 10^8^ viral particles (vp)/µl) showed viral transduction by visualization of GFP at week 5 after subretinal injections, whereas PBS-injected eyes showed no GFP (Figure 2a). As further confirmation of viral transduction, GFP-positive eyes were harvested, and RPE/choroidal cryosections were immunostained for GFP. GFP colocalized with RPE65-labeled RPE cells (Figure 2b). Also, there was increased Rap1 protein in RPE/choroidal lysates from scAAV2-CARap1a at the 5 × 10^8^ vp/µl dose compared to scAAV2-Con or PBS injection (Figure 2c).

To then determine if the dose affected the structure or visual function of the retina, optical coherence tomography (OCT) and focal electroretinography (fERG) were performed. At the points of injection in all groups, there were changes in retinal structure with loss of the inner segment/outer segment lines and photoreceptors (Figure 2d, upper row). Outside the injection site but within the regions where subretinal injections of scAAV2-CARap1a or scAAV2-Con had been delivered 5 weeks earlier, there were no morphological differences noted by OCT (Figure 2d, lower row). No differences in retinal structure by OCT were noted between GFP+ or GFP- regions within the same eyes (Figure 2d, upper and lower rows, respectively). There were also no differences in retinal structure in PBS-, scAAV2-Con, or scAAV-CARap1a (Figure 2e) or in amplitudes of a-waves and b-waves by fERG (Figure 2f). Therefore, both scAAV2s effectively and safely transduced RPE cells 5 weeks after subretinal injections, and scAAV2-CARap1a increased RPE Rap1 protein compared to scAAV2-Con. Therefore, these conditions were used in subsequent experiments.

### Expression of active Rap1a in RPE cells reduces CNV in Rap1b-deficient mice

**Figure 3:**
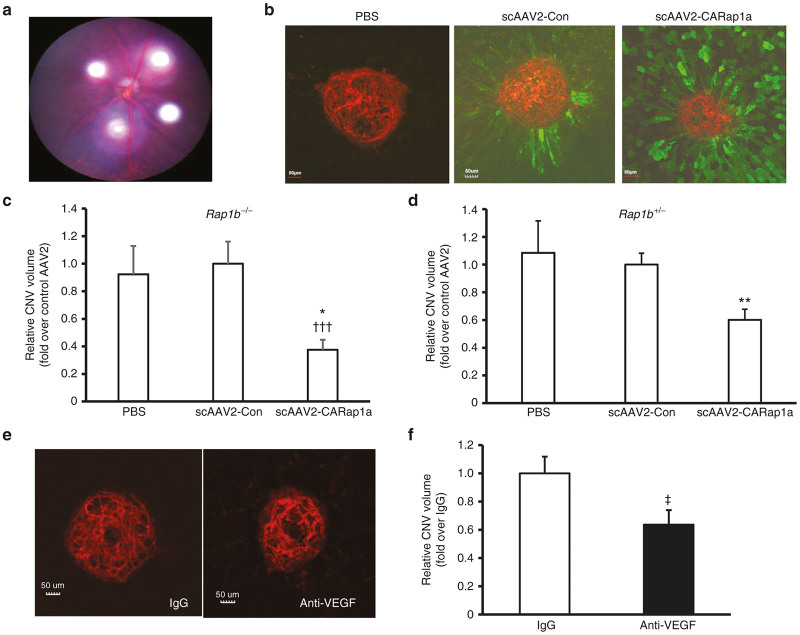
Expression of active Rap1a in RPE cells reduces choroidal neovascularization (CNV) in Rap1b deficient mice. (**a**) Micron IV imaging of laser spots in the retina of mice in the laser-induced CNV model; (**b**) Images of retinal pigment epithelial (RPE)/choroidal flat mounts stained with isolectin and anti-GFP antibody and normalized CNV volume (fold change over scAAV2-Con) in (**c**) *Rap1b*^*-/-*^ and (**d**) *Rap1b*^*+/-*^ mice treated with subretinal injections of PBS, control virus scAAV2-Con or scAAV2-CARap1a virus at 5X10^8^ vp/µl (*P < 0.05, **P < 0.01 versus scAAV2-Con; ^†††^*P*<0.001 versus PBS); (**e**) Images of RPE/choroidal flat mounts stained with isolectin and (**f**) normalized CNV volume (fold change over IgG) of *Rap1b*^*+/-*^ mice treated with intravitreal injections of either isotype IgG or a neutralizing antibody to mouse VEGF164 (anti-VEGF) (^‡^*P* < 0.05 versus IgG).

As Rap1b is implicated in promoting VEGF/VEGFR2-mediated angiogenesis,^15,16^ which is important in neovascular AMD,^18,19^ we restricted activation of Rap1 to only the Rap1a isoform by using *Rap1b*^*-/-*^ mice. We previously published that activation of Rap1a with the chemical, 8-CPT-2Me-cAMP, delivered as an intravitreal injection, reduced CNV in *Rap1b*^*-/-*^ mice.^14^ However, here we wished to focus on the hypothesis that activation of Rap1a specifically in RPE cells would inhibit CNV and wished to eliminate effects from activation of Rap1a in CECs or other cells. We, therefore, introduced activated Rap1a specifically into RPE cells by subretinal injection of scAAV2-CARap1a and compared outcomes to scAAV2-Con or PBS-injected eyes. Five weeks after subretinal injections of scAAV2 vectors or PBS, *Rap1b*^*-/-*^ and *Rap1b*^*+/-*^ mice were treated with laser to induce CNV. As shown in Figure 3a, four laser spots were distributed in the retina, each about 2 disc diameters from the optic nerve. One week after laser treatment, eyes were harvested for RPE/choroidal flat mounts, and isolectin-stained CNV spots within regions of GFP-positive RPE cells were analyzed for CNV volume (Figure 3b). As shown in Figure 3c,d, eyes injected with scAAV2-CARap1a had significantly reduced CNV volume with 60% reduction in *Rap1b*^*-/-*^ (Figure 3c) and 40% reduction in *Rap1b*^*+/-*^ mice (Figure 3d) compared to either PBS or scAAV2-Con injections. There was no significant effect on CNV of scAAV2-CARap1a injected into *Rap1b*^*+/+*^ mice compared to scAAV2-Con or PBS in part due variability of CNV lesions, potentially due to different isoform dominance and activation (data not shown). scAAV2-CARap1a did not increase apoptosis either within CNV lesions or in the retina overlying the CNV lesions compared to eyes that had been injected with either PBS or scAAV2-Con (data not shown). As a comparison reflecting the current standard of care for neovascular AMD, an intravitreal injection of a neutralizing antibody against mouse VEGF_164_, the most prevalent VEGF splice variant, was delivered into both eyes of a separate group of 11-week-old *Rap1b*^*+/-*^ mice immediately after laser treatment and compared to similar *Rap1b*^*+/-*^ mice that received intravitreal control isotype IgG. One week later, CNV volumes analyzed in isolectin stained RPE/choroidal flatmounts (Figure 3e) showed that anti-VEGF caused a 36% reduction in CNV volume compared to IgG control (Figure 3f), comparable to the effects seen from activation of Rap1a specifically in RPE cells in *Rap1b*^*+/-*^ mice.

### Expression of active Rap1a in RPE cells has no effect on VEGF-mediated signaling, but increases junctional cadherins in RPE/choroids

**Figure 4:**
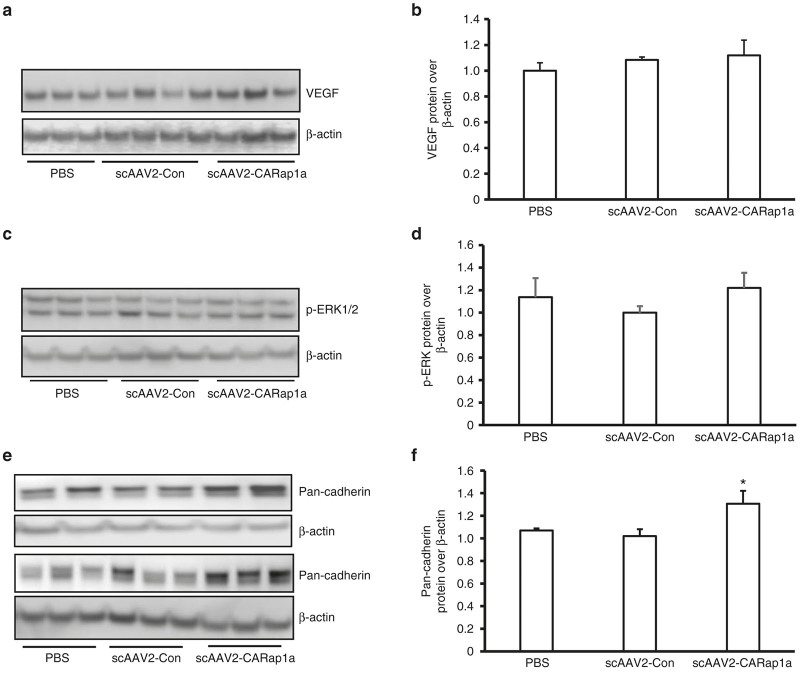
Expression of active Rap1a in RPE cells has no effect on VEGF, but increases junctional protein, cadherin, in RPE/choroids. Western blots of (**a** and **b**) VEGF protein, (**c** and **d**) phosphorylation of ERK1/2 (p-ERK1/2) and (**e** and **f**) pan-cadherin in retinal pigment epithelial (RPE)/choroids from *Rap1b*^*+/-*^ mice treated with subretinal injection of PBS, scAAV2-Con or scAAV2-CARap1a (**a, c** and **e** are representative gels and **b, d** and **f** are densitometry of gels; *P < 0.05 versus scAAV2-Con).

VEGF signaling plays an important role in human neovascular AMD.^18,19^ Since the fold decrease in CNV volume in scAAV2-CARap1a versus scAAV2-Con was comparable to that from intravitreal anti-VEGF versus IgG, we wished to determine if activation of Rap1a affected VEGF expression or signaling. We previously found that laser-induced CNV increased NADPH oxidase-generated ROS in CNV^20^ and VEGF protein in RPE/choroidal lysates in wild-type C57Bl/6 mice.^21^ By western blot analysis, VEGF protein and phosphorylation of ERK (p-ERK), a downstream signaling effector of activated VEGFR2, were measured in RPE/choroids from PBS, scAAV2-Con- or scAAV2-CARap1a-treated and lasered *Rap1b*^*+/-*^ mice. There were no differences in VEGF protein concentration (Figure 4a,b) or p-ERK1/2 (Y204) (Figure 4c,d) in RPE/choroids among any groups. In the same tissue lysates, however, the junctional cadherin proteins were significantly increased by scAAV2-CARap1a compared to scAAV2-Con treatment (Figure 4e,f). We used a pan-cadherin antibody to identify both E-cadherin, which is generally more abundant in epithelial cells, and also N-cadherin, which is more abundant in RPE cells.^22^

### Expression of active Rap1a maintains RPE barrier function following TNFα stimulation through an IQGAP1-mediated interaction with β-catenin

**Figure 5:**
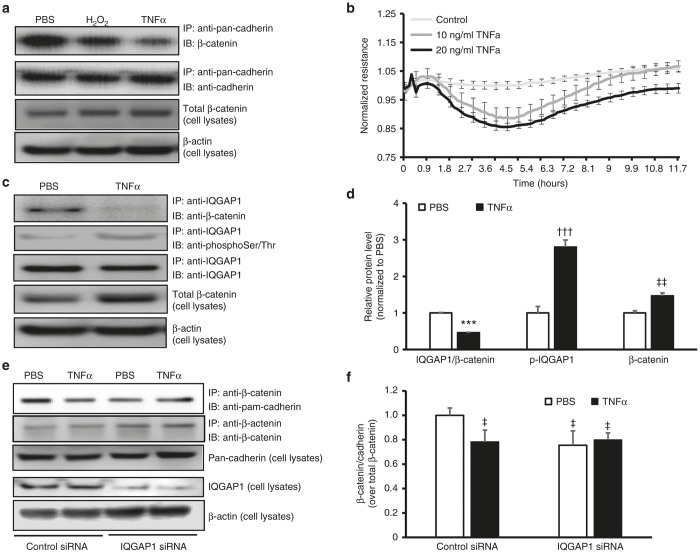
TNFα reduces RPE barrier integrity by a mechanism involving IQGAP1 interaction with β-catenin and phosphorylation. (**a**) Co-immunoprecipitation of junctional protein pan-cadherin and β-catenin and western blots of β-catenin and β-actin in retinal pigment epithelial (RPE) cells exposed to PBS, H_2_O_2_ (10 µmol/l), or human recombinant TNFα (20 ng/ml) overnight; (**b**) Electric Cell-substrate Impedance Sensing (ECIS) analysis of barrier resistance of RPE cells exposed to human recombinant TNFα (10 or 20 ng/ml) or control PBS for 12 hours; (**c**) and (**d**) coimmunoprecipitation of IQGAP1 and β-catenin or phosphorylation of IQGAP1 and western blots of β-catenin and β-actin in RPE cells exposed to TNFα (20 ng/ml) or control PBS for 5 hours (**c**, representative gel images and **d**, quantification of densitometry of C; ***P < 0.001 versus PBS of IQGAP1/β-catenin; ^†††^*P* < 0.001 versus PBS of p-IQGAP1; ^‡‡^*P* < 0.01 versus PBS of β-catenin) and (**e**) and (**f**) coimmunoprecipitation of β-catenin and pan-cadherin and western blot of IQGAP1 and pan-cadherin in RPE cells transfected with IQGAP1 siRNA or control siRNA and treated with TNFα (20 ng/ml) or control PBS for 5 hours (**e**, representative gel images and **f**, quantification of densitometry of **e**; ^‡^*P* < 0.05 versus Control siRNA/PBS).

We^23^ and others^24^ reported that TNFα contributes to CNV induced by laser in murine eyes, at least in part, by activating NADPH oxidase in RPE cells.^23^ We now wanted to know if this TNFα-induced ROS generation provoked RPE barrier compromise. RPE barrier integrity was determined two ways: formation of junctional pan-cadherin/β-catenin complexes by coimmunoprecipitation (CO-IP) and electric cell-substrate impedance sensing (ECIS) analysis. Human RPE cells were cultured until a monolayer was formed. Cells were then exposed to PBS or TNFα (20 ng/ml) overnight. H_2_O_2_ (10 µmol/l) was used as a positive control.^14^ Compared to PBS, TNFα decreased CO-IP of pan-cadherin/β-catenin to a similar level as H_2_O_2_; however, the β-catenin protein in cell lysates was not reduced by either TNFα or H_2_O_2_ treatment (Figure 5a). In a parallel experiment, RPE cells were plated into electrode coated ECIS culture-ware and maintained until electrical resistance of RPE cells was stabilized. Human RPE cells were then exposed to TNFα or PBS overnight, and resistance was monitored. As shown in Figure 5b, compared to PBS, electrical resistance of RPE cells started to decrease 1 hour after cells were exposed to TNFα and was maximally reduced after 5 hours of TNFα-incubation (Figure 5b).

We next studied the mechanism involved in TNFα-mediated reduced RPE barrier resistance. We are interested in the multi-domain scaffold protein, IQGAP1, which regulates cell adhesion and cell motility by interacting with F-actin, cadherins and β-catenin.^25–28^ In the unphosphorylated or unbound state, IQGAP1 does not play a role in many signaling events. However, when phosphorylated, IQGAP1 can facilitate pathologic signaling.^29^ A role of IQGAP1 in enhancing barrier function has been proposed;^30,31^ however, its role in RPE barrier integrity is unknown. We hypothesized that the interaction between IQGAP1 and β-catenin facilitated junctional cadherin proteins and β-catenin association, and thereby maintained barrier integrity of the RPE cells. To test this hypothesis, CO-IP of IQGAP1 and β-catenin was performed in RPE cells treated with TNFα vs. control. As shown in Figure 5c,d, after 5 hours of TNFα exposure, CO-IP of IQGAP1 and β-catenin was significantly decreased. This decreased interaction of β-catenin with IQGAP1 was not a result of reduced β-catenin in RPE cells, as β-catenin protein was increased in RPE cells treated with TNFα (Figure 3d), a finding consistent with our previous study.^21^ In the same lysates, there was increased serine/threonine phosphorylation of IQGAP1. As a further confirmation, RPE barrier integrity was assessed by CO-IP of pan-cadherin and β-catenin in RPE cells transfected with IQGAP1 siRNA or control siRNA and treated with TNFα. As shown in Figure 5e,f, in control siRNA-transfected RPE cells, compared to PBS, TNFα treatment significantly decreased CO-IP of β-catenin and pan-cadherin, analogous to that seen in Figure 5a. However, the decreased interaction of β-catenin and pan-cadherin induced by TNFα treatment was not seen in RPE cells knocked down for IQGAP1 when compared to control siRNA (Figure 5f). Neither TNFα treatment nor IQGAP1 knockdown affected pan-cadherin protein levels in RPE cells. Altogether, the data shown in Figure 5 suggest that TNFα reduces RPE barrier integrity through a mechanism involving IQGAP1 phosphorylation and interaction with β-catenin.

**Figure 6:**
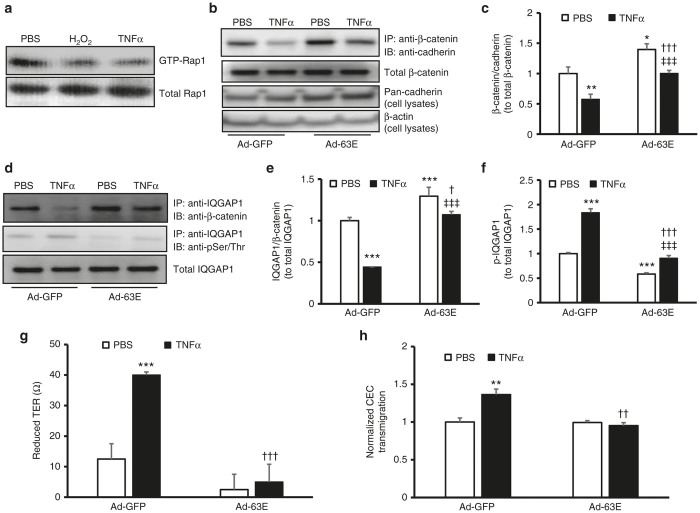
Expression of active Rap1a in RPE cells reduces cell barrier compromise from TNFα by modulating IQGAP1 interactions with β-catenin and IQGAP1 phosphorylation. Rap1 activity assay in (**a**) retinal pigment epithelial (RPE) cells exposed to PBS, H_2_O_2_ (10 µmol/l), or human recombinant TNFα (20 ng/ml) overnight and (**b**) and (**c**) coimmunoprecipitation of β-catenin and pan-cadherin and western blot of pan-cadherin (**b**, representative gel images and **c**, quantification of densitometry of **b**; fold change over Ad-GFP/PBS) and (**d–f**) coimmunoprecipitation of IQGAP1 and β-catenin and phosphorylation of IQGAP1 in RPE cells transduced with Ad-GFP or Ad-63E and treated with PBS or TNFα (20 ng/ml) for 5 hours (d, representative gel images and **e** and **f**, quantification of densitometry of d) (**g**) Transepithelial resistance (TER) and (**h**) normalized CEC transmigration (fold change over Ad-GFP/PBS) of RPE cells transduced with Ad-GFP or Ad-63E and treated with PBS or TNFα (20 ng/ml) for 24 hours (*P < 0.05, **P < 0.01 and ***P < 0.001 versus Ad-GFP/PBS; ^†^*P* < 0.05, ^††^*P* < 0.01 and ^†††^*P* < 0.001 versus Ad-GFP/TNFα and ^‡‡‡^*P* < 0.001 versus Ad-63E/PBS).

We previously reported that activation of Rap1 increases RPE barrier integrity.^9,14^ We found TNFα also reduced active Rap1 in human RPE cells (Figure 6a). To increase active Rap1a, cultured RPE cells were transduced with adenovirus expressing the Rap1a Q63E mutant that constitutively activates Rap1a (Ad-63E) or adenovirus expressing only GFP (Ad-GFP) as a control. Forty-eight hours after viral transduction, RPE cells were exposed to PBS or TNFα for 5 hours and CO-IP of β-catenin and cadherin was determined in RPE cells. As shown in Figure 6b,c, compared to Ad-GFP and PBS treatment, RPE cells transduced with Ad-63E had significantly increased CO-IP of β-catenin and pan-cadherin, suggesting greater structural integrity of the junctions. In Ad-GFP-transduced RPE cells, TNFα treatment significantly decreased CO-IP of β-catenin and pan-cadherin, and this reduction was significantly inhibited by active Rap1a in RPE cells transduced with Ad-63E (Figure 6c). Ad-63E also increased cadherin protein level (Figure 6b), similar to that seen in RPE/choroids from mouse eyes treated with scAAV2-CARap1a (Figure 4e,f). Furthermore, the reduced CO-IP of IQGAP1/β-catenin (Figure 6d,e), and increased phosphorylation of IQGAP1 from TNFα treatment (Figure 6d,f) were inhibited by Ad-63E transduction. Altogether, the results support the line of thinking that active Rap1a maintains RPE barrier integrity after exposure to TNFα by modulating the interaction between IQGAP1 and β-catenin in a phosphorylation-dependent mechanism.

We also determined the effect of active Rap1a on RPE barrier function by measuring transepithelial electrical resistance (TER) in the presence of increased active Rap1a compared to control. TERs of human RPE monolayers transduced with Ad-GFP and Ad-63E were measured prior to and following overnight treatment with TNFα, and the differences in TER were then calculated. As shown in Figure 6g, TNFα mediated a reduction in TER that was inhibited in Ad-63E-transduced RPE cells compared to Ad-GFP, suggesting that active Rap1a was sufficient to protect against RPE barrier compromise from TNFα exposure. Finally, we also found that expression of active Rap1a in Ad-63E-transduced RPE cells was sufficient to inhibit CEC transmigration in the human RPE cell-CEC transmigration assay (Figure 6h).

## Discussion

Intact RPE barrier integrity is important for the health and function of the retinal photoreceptors and choriocapillaris.^32^ In neovascular AMD, severe vision loss occurs when the RPE barrier is compromised and when activated CECs are able to migrate across the RPE monolayer into the sensory retina and develop into leaky CNV. Increasing RPE barrier integrity may be one way to prevent or reduce CNV. We previously reported that activation of the Rap1a and 1b isoforms enhance RPE barrier integrity in different ways.^9,14^ However, active Rap1b also induces angiogenesis,^15^ so broad activation of both, closely related, isoforms may be counterproductive, depending on the prevalence and activation of Rap1 isoforms, when attempting to inhibit CNV. We also previously reported that activation of Rap1a in *Rap1b*^*-/-*^ mice by injection of intravitreal 8-CPT-2Me-cAMP reduced CNV.^14^ Here, we wished to test the hypothesis that activation of Rap1a specifically in RPE cells would enhance RPE barrier integrity and be sufficient to reduce CNV.

To increase Rap1a activity in RPE cells, a constitutively active Rap1a mutant was engineered into a self-complementary adeno-associated virus 2 vector with a GFP tag driven by a murine RPE65 promoter and was shown to specifically target the RPE cells (Figure 1b). *In vivo* Micron IV live imaging demonstrated GFP expression after injection of 5 × 10^8^ vp/µl showing effective transduction of the mouse RPE cells at 5 weeks after subretinal administration. Western blots confirmed that scAAV2-CARap1a increased Rap1 protein level in RPE cells compared to scAAV2-Con. In wild-type C57Bl/6 mice, subretinal injections of scAAV2-Rap1a did not reduce CNV compared to controls. This may have been in part due to inability to control for variable expression and state of activation of each isoform in wild type RPE cells. Our *in vitro* data supported the role of active Rap1a in reducing NADPH oxidase-generated ROS in RPE cells and in improving RPE barrier integrity.^14^ We speculate that Rap1b is abundantly expressed in mice and may mask the effects from introducing constitutively active Rap1a. For example, *Rap1b*^*-/-*^ produce larger CNV following laser injury than did *Rap1b*^*+/+*^.^9^ Therefore, to eliminate Rap1b, we used *Rap1b*^*-/-*^ mice to test the effects of increased active Rap1a specifically in RPE cells. We also treated *Rap1b*^*+/-*^ mice, anticipating the heterozygote with some Rap1b in all cells might have a reduced inhibitory effect on CNV from active Rap1a, because Rap1b is involved in angiogenesis.^15^ We found that scAAV2-CARap1a significantly reduced CNV in both *Rap1b*^*-/-*^ and *Rap1b*^*+/-*^ mice with a greater fold decrease in the homozygous knockout mice treated with scAAV2-CARap1a compared to control scAAV2-Con, supporting this hypothesis. The introduction of CARap1a specifically into RPE reduced CNV in the Rap1b null mice but not in wild-type mice. We are pursuing the hypotheses that dominance of isoforms and relative ratio of activated vs. inactivated isoforms differs across species and tissues to partially account for this. It may also be that Rap1a activation in CECs in addition to activation in RPE is needed to fully interfere with CNV.^33^

scAAV2-CARap1a did not increase retinal apoptosis. Compared to PBS, ocular injections of either of scAAV2 vectors did not cause adverse effects on retinal morphology determined by OCT imaging or visual function by focal ERG. Taken together, these results support the line of thinking that increased Rap1a in RPE cells can effectively reduce CNV, and ocular injection of scAAV2 driven by an RPE65 promoter can efficiently target the RPE cells without causing structural or functional deficits of the retina.

Since both inflammatory and angiogenic pathways are important in the development of neovascular AMD,^34–36^ ligands from each can act to cause a feed-forward loop to overwhelm homeostasis and enable CEC migration and transmigration of the RPE cells,^6^ an essential step in the formation of CNV in the sensory retina in neovascular AMD.^10^ We previously reported that the proinflammatory cytokine, TNFα, was increased in RPE/choroids from mice that were treated with laser to induce CNV.^21^ Here, we determined that TNFα reduced RPE barrier resistance, as evidenced by decreased junctional pan-cadherin/β-catenin complexes and transepithelial resistance. TNFα-induced RPE barrier compromise was associated with decreased interaction of junctional protein β-catenin with the scaffold protein, IQGAP1. IQGAP1 is a multidomain protein that is believed to integrate multiple intracellular signaling pathways involved in cell adhesion and cell motility by interacting with partner proteins.^37^ At its RasGAP-like C-terminal binding domain, IQGAP1 interacts with cadherin proteins and β-catenin. Therefore, we tested the hypothesis that IQGAP1 facilitated the formation of junctional protein complexes involving cadherins and β-catenin, and, thereby, protected RPE barrier compromise from TNFα. We found that knockdown of IQGAP1 by siRNA transfection in RPE cells reduced cadherin and β-catenin complexes but also inhibited the TNFα-induced decrease in cadherin and β-catenin complexes. This suggested that when IQGAP1 is unphosphorylated, it interacts with β-catenin and brings together β-catenin and cadherin for RPE junctional integrity. TNFα-induced phosphorylation of IQGAP1 interferes with interactions between β-catenin and cadherins, which are involved in RPE junctional function. We further found that increased active Rap1a in RPE cells inhibited TNFα-mediated reduction in cadherin and β-catenin complexes and IQGAP1 and β-catenin interactions. These findings were associated with increased TER, reduced TNFα-induced IQGAP1 phosphorylation, and reduced CEC transmigration of the RPE monolayer.

We previously reported that VEGF protein was increased in RPE cell/choroids from wild type C57Bl/6 mice treated with laser 7 days earlier.^21^ Here, however, in the condition of partial knockdown of Rap1b, we did not find that scAAV2-CARap1a modulated VEGF or VEGF signaling compared to scAAV2-Con in *Rap1b* heterozygous knockout mice. Repeated delivery of anti-VEGF agents by intravitreal injection is the standard of care for neovascular AMD. However, clinical studies show that this anti-VEGF treatment improves outcomes in only about 40% of cases.^1^ In addition to potential complications from repeated intravitreal injection, there are also concerns that concomitant removal of the beneficial effects of VEGF will potentiate atrophy of the choriocapillaris and retinal elements in AMD patients.^38,39^ Therefore, a means to increase RPE barrier integrity, potentially by regulating active Rap1, may improve outcomes in neovascular AMD and reduce the number of anti-VEGF injections.

In summary, we generated scAAV2 vectors to deliver active Rap1a that targeted and increased Rap1a in RPE cells. The data provide evidence for the first time that increased active Rap1a specifically in the RPE layer can effectively increase RPE barrier integrity and reduce laser-induced CNV in Rap1b deficient mice without adverse effects on retinal structure and function. We also present a novel mechanism by which activated Rap1a specifically in RPE cells can counteract TNFα-induced RPE barrier compromise by regulating the phosphorylation of the scaffold protein, IQGAP1, and its interaction with the junctional protein β-catenin. Currently, effective inhibition of CNV by active Rap1a in RPE is seen in Rap1b null mice. More study is warranted.

## Materials and Methods

### Animals

*Rap1b*^-/-^, *Rap1b*^*+/-*^ and littermate wild-type mice were generated as described previously.^40^ Six- to 12-week-old mice were used in these studies. All animal procedures were performed in accordance with the University of Utah guidelines (Guide for the Care and Use of Laboratory Animals) and the Association for Research in Vision and Ophthalmology Statement for the Use of Animals in Ophthalmic and Vision Research. Anesthesia was obtained with ketamine (100 mg/kg) and xylazine (20 mg/kg), and euthanasia was performed under CO_2_ exposure. All efforts were made to minimize suffering.

### Construction of RPE-65 promoter driven self-complementary adeno-associated virus 2

A self-complementary adeno-associated virus 2 (scAAV2) vector was purchased from University of North Carolina Vector Core (Chapel Hill, NC). The scAAV2 vectors contain sequences for GFP. The CMV promoter was replaced with a murine RPE65 promotor (kindly provided by T. Michael Redwood), and synthetic sequence for constitutively active human Rap1a Q63E mutant (CARap1a) was then cloned into the scAAV2 vector with the RPE65 promoter (scAAV2-RPE65-CARap1a-GFP). The scAAV2 construct without CARap1a was used as a control vector (scAAV2-RPE65-GFP). Sequences of plasmid DNAs, scAAV2-RPE-65-CARap1a-GFP and scAAV2-RPE65-GFP were confirmed by DNA sequencing (HSC Core Facility at University of Utah, Salt Lake City, UT). Viruses were produced, purified and titered at the UNC Vector Core.

### Ocular injections, micron IV imaging, optical coherence tomography, focal electroretinography and laser-induced CNV model

One microliter containing scAAV2 at 5 × 10^8^ viral particles or PBS was injected into the subretinal space of each eye of 6-week-old mice. The scAAV2 viral transduction was monitored by *in vivo* live imaging using the Micron IV retinal imaging system (Phoenix Research Laboratories, Pleasanton, CA) under a GFP filter. Image-guided spectral domain coherence tomography (OCT, Phoenix Labs, Manchester, CT) was used to visualize retinal structure and morphology after AAV2 viral injections.

Four weeks after scAAV2 vector injection, retinal function was assessed by image guided focal electroretinogram (fERG) using Micron IV technology. Mice were fully dark adapted overnight and subsequently handled under dim red light illumination. After anesthesia and pupil dilation with tropicamide (1%) eye drops (Bausch and Lomb, Tampa, FL), mice were kept on a heat pad throughout the procedure to maintain constant body temperature. Subdermal needle electrodes were inserted underneath the skin at the base of the tail (ground electrode) and between the eyes on the forehead (reference electrode). Corneas were lubricated with coupling gel (GenTeal, hypromellose 0.3%, Alcon, Fort Worth, TX), the objective/electrode was advanced near the corneal surface, deep red illumination was used to focus on the retina and an unlasered retinal section of 0.25 mm diameter (spot size A) was chosen. Responses to focal light stimuli were elicited at luminances ranging from 1.1 to 3.8 cd s m^−2^ with 30 sweeps at 2 seconds intervals. Data is displayed as the mean amplitude (in µV) of the a-wave (as a measure of photoreceptor function) and b-wave (as a measure of bipolar cell function).

Five weeks after AAV2 viral injection, 11-week-old mice received laser to induce CNV. Both eyes of each mouse were dilated with one drop of 1% tropicamide ophthalmic solution. After dilation, mice were anesthetized, placed onto a heated stage and treated with 4–5 spots of 532 nm laser photocoagulation each about 2 disc diameters from the optic nerve with the Phoenix Image-Guided Laser System 94 (Phoenix Micron IV, Pleasanton, CA) at settings of ~400 mW intensity and 100 ms duration. Adequate treatment was assessed by cavitation bubbles confirming disruption of Bruch’s membrane.

For anti-VEGF treatment, 11-week-old mice received bilateral intravitreal injections of 1 μl of a neutralizing antibody to mouse VEGF or of isotype IgG (50 ng, R&D Systems, Minneapolis, MN) immediately following laser injury to mimic current standard of care for human neovascular AMD. Seven days after laser treatment, mice were euthanized, and eyes were collected for the analysis of CNV volume and protein analysis.

### Analysis of lesion volume in RPE/Choroid flat mounts

Eyes were fixed in 4% paraformaldehyde (Electron Microscopy Sciences, Hatfield, PA) for 2 hours after removal of the cornea and lens. Posterior eyecups of the RPE/choroid/sclera were dissected, and the vitreous was removed. Eyecups were incubated overnight at 4 °C with AlexaFluor 647-conjugated Isolectin B4 (1:200, Invitrogen, Carlsbad, CA) to label invading choroidal vessels and anti-GFP antibody to label GFP in RPE cells (1:500, ABCAM, Cambridge, MA). After staining, the eyecups were flattened by cutting radial incisions and flatmounted onto a microscope slide with vectashield mounting medium (Vector Laboratories, Burlingame, CA) for confocal imaging. Flatmounts were imaged by taking optical Z-sections at 3 µm increments with a confocal microscope (FV1000, Olympus, Japan), and area for each Z-section was measured manually using Image J and summed to create a volume. Only lesions with GFP-positive RPE cells were included for CNV volume analysis. Lesions with obvious hemorrhage or bridging CNV were excluded. Images were measured and confirmed by two masked reviewers.

### Immunostaining in retinal cryosections

Eyes were fixed in 4% paraformaldehyde (Electron Microscopy Sciences, Hatfield, PA) for 2 hours after removal of cornea and lens, followed by incubation with 10 to 30% sucrose overnight at 4 °C. Eye cups were then embedded in optimal cutting temperature (OCT) (Tissue Tek, Hatfield, PA) and sectioned. For immunofluorescence, cryosections (12 μm) were first incubated in 5% normal goat serum in PBS/0.1% TritonX-100 for 1 hour to block nonspecific binding of the primary antibody (see Table 1). Sections were incubated with rabbit anti-GFP (1:200) and RPE65 (1:100, ABCAM) overnight at 4 °C. After three washes in PBS, sections were incubated for 1 hour with a 1:200 dilution of FITC-conjugated goat anti-rabbit secondary antibody (Invitrogen, Carlsbad, CA) for GFP and AlexaFluor 594-conjugated goat anti-mouse secondary antibody for RPE65. The sections were rinsed in PBS and mounted in DAPI-Fluoromount-G (SouthernBiotech, Birmingham, AL). Images were captured with an inverted microscope (OLYMPUS 1X81: Japan) at 20× magnification.

### Cell culture and transduction of RPE with adenovirus and treatments

Human primary RPE (RPE) cells (Lonza, Walkersville, MD) were grown in RPE cell growth media (RtEGM, Lonza) with 2% FBS and used from passage 3–5. Human primary choroidal endothelial cells (CECs) were isolated from donor eyes obtained from Utah Eye bank (Salt Lake City, UT) as previously described.^41^ CECs were grown in endothelial growth media (EGM2, Lonza) supplemented with 5% FBS and used from passage 1–5.

RPE cells were transduced with adenoviral constructs expressing GFP (Ad-GFP) or active Rap1a (Ad-63E). Forty-eight to 72 hours later when 90% of RPE cells were GFP positive, cells were incubated with recombinant human TNFα (10–20 ng/ml, R&D Systems), H_2_O_2_ (10 µmol/l) or PBS control for 5 hours or overnight. Cells were then collected for western blots, coimmunoprecipitation (CO-IP) and Rap1 activity assay.

### Immunoprecipitation and Immunoblots

Please see Table 1 for a list of antibodies and catalog numbers used. RPE/choroidal tissues and RPE cells were lysed in radio immunoprecipitation assay buffer (RIPA) (20 mmol/l Tris pH 7.4, 120 mmol/l NaCl, 0.5% sodium deoxycholic acid, 1% Triton X-100, 0.1% SDS, 10% glycerol) with protease inhibitor cocktail (Roche Diagnostics, Indianapolis, IN) and phosphatase inhibitor orthovanadate (2 mmol/l, Sigma-Aldrich, St. Louis, MO). Lysates were centrifuged at 13,000 rpm for 5 minutes at 4 °C. Protein concentration in the supernatant was quantified by bicinchoninic acid assay (BCA) (Pierce, Rockford, IL). Thirty micrograms of protein from RPE/choroids were loaded into 4 to 12% NuPAGE Bis-Tris gels (Invitrogen, Carlsbad, CA), and transferred to a PVDF membrane (Invitrogen), incubated with antibodies to VEGF and phosphorylated ERK (1:500, Santa Cruz Biotechnology, Santa Cruz, CA), and Rap1 and pan-cadherin (1:1,000, Cell signaling Technology, Danvers, MA) overnight at 4 °C.

For coimmunoprecipitation (CO-IP), total protein (500 µg in 500 ml) for each sample was incubated with antibody to β-catenin (1:100, Cell signaling Technology), IQGAP1 (1:100, BD Transduction Laboratories, Franklin Lakes, NJ) or pan-cadherin (1:100, Cell signaling Technology) by gently rocking at 4 °C overnight with 10 µl of Dynabeads protein G (Invitrogen). The antibody/protein/agarose complex was washed three times with RIPA buffer and resuspended in 2× sample buffer. Protein complex was separated by NuPAGE 4 to 12% Bis-Tris Gels (Invitrogen) and transferred to a PVDF membrane and then incubated with antibodies to pan-cadherin (1:1,000) for CO-IP of β-catenin/cadherin, β-catenin (1:1,000) for CO-IP of pan-cadherin/β-catenin or CO-IP of IQGAP1/β-catenin, or phosphoserine/threonine (1:1,000, BD Transduction Laboratories) for phosphorylated IQGAP1 overnight at 4 °C. All membranes were reprobed with anti-β-catenin, anti-IQGAP1 or anti-pan-cadherin to ensure equal β-catenin, IQGAP1 or pan-cadherin protein loading.

For Rap1 activity assay in RPE cells, 300 µg of protein from each treatment was incubated with anti-GTP-Rap1 antibody (NewEast, Malvern, PA) and Dynabeads protein G at 4 °C overnight. Bead-bound active Rap1 was determined by western blots using a monoclonal antibody against Rap1 (BD Transduction Laboratories).

Densitometry analysis was done with the use of the software UN-SCAN-IT version 6.1 (Silk Scientific, Orem, UT).

### ECIS assay and TER of RPE barrier integrity

Transepithelial resistance was measured by either the electric cell-substrate impedance sensing (ECIS)-Zθ system (Applied Biophysics, Troy, NY) or the EOVM^2^ system with the EndOhm electrode (World precision Instruments, Sarasota FL). For ECIS, an electrode culture assay was coated with attachment factor protein (Invitrogen). RPE cells, 50,000 per well, were seeded in complete media onto the electrode culture assay and monitored until a stable monolayer formed. Cells were then treated with 10 or 20 ng/ml human recombinant TNFα for 12 hours. Resistance across the monolayer was measured with 40 electrodes per well (1,000–2,000 cells) using the ECIS.

For TER measurement with the EOVM^2^ system, RPE cells were cultured in Transwells with 0.4 µm pores (Corning Incorporated, Corning, NY) until cells formed a monolayer and had TER measurements > 100 ohms.cm^2^. RPE cells were then transduced with adenoviral construct Ad-63E or control virus Ad-GFP. Forty-eight hours after virus transduction, RPE cells were incubated with human recombinant TNFα (20 ng/ml). TER of RPE monolayer was measured prior to addition of TNFα and 24 hours after incubation with TNFα. Data are representative of at least three independent experiments.

### CEC transmigration assays

Transmigration of CECs across the RPE monolayer was measured as previously described.^41^ RPE cells were first plated onto the underside of Transwells having 8 µm pores (Corning Incorporated) until a monolayer was formed. The cells were then transduced with adenoviral construct Ad-63E or control virus Ad-GFP for 48 hours. CECs labeled with fluorescent dye (Vybrant Dil Cell-labeling solution; Invitrogen) were plated into the inserts with addition of recombinant TNFα (20 ng/ml) in the inserts and wells. Twenty-four hours after adding the CECs, migrated CECs were counted using fluorescent microscopy.

### Statistical analysis

Significant differences between groups were determined by analysis of variance with *post hoc* protected Bonferroni Multiple Comparison Test. Results were displayed as Mean ± standard error of the mean. A *P* value of <0.05 was considered statistically significant. For animal studies, at least five individual mice and bilateral choroidal flat mounts were analyzed for CNV volume (*n* = 4 spots/eye), Micron IV imaging and optical coherence tomography. The results of the electroretinography analysis included an average of three eyes from three individual mice. Retinal sections for GFP staining and western blots of Rap1 protein were taken from five to seven different mice. For *in vitro* studies, each experimental condition included an *n* = 6–9 from three independent experiments.

## Figures and Tables

**Table 1:** List of antibodies used in this study

	*Antibody name*	*Company*	*Catalog number*
1	GFP	Abcam	ab290
2	RPE65	Abcam	ab78036
3	Rap1	BD Transduction Laboratories	610195
4	β-actin	Santa Cruz Biotechnology	sc-47778
5	VEGF	Santa Cruz Biotechnology	sc-7269
6	phospho-ERK1/2	Santa Cruz Biotechnology	sc-7383
7	pan-cadherin	Cell Signaling Technology	4073
8	β-catenin	Cell Signaling Technology	8480
9	IQGAP1	BD Transduction Laboratories	610611
10	Phosphoserine/Threonine	BD Transduction Laboratories	612548
11	GTP-Rap1	NewEast Biosciences	26912
